# Predictors of early death in a cohort of Ethiopian patients treated with HAART

**DOI:** 10.1186/1471-2334-6-136

**Published:** 2006-09-01

**Authors:** Degu Jerene, Aschalew Endale, Yewubnesh Hailu, Bernt Lindtjørn

**Affiliations:** 1 Centre for International Health, University of Bergen, Bergen, Norway; 2 Arba Minch Hospital, Arba Minch, Ethiopia

## Abstract

**Background:**

HAART has improved the survival of HIV infected patients. However, compared to patients in high-income countries, patients in resource-poor countries have higher mortality rates. Our objective was to identify independent risk factors for death in Ethiopian patients treated with HAART.

**Methods:**

In a district hospital in Ethiopia, we treated adult HIV infected patients with HAART based on clinical and total lymphocyte count (TLC) criteria. We measured body weight and complete blood cell count at baseline, 4 weeks later, then repeated weight every month and complete blood cell count every 12 weeks. Time to death was the main outcome variable. We used the Kaplan Meier and Cox regression survival analyses to identify prognostic markers. Also, we calculated mortality rates for the different phases of the follow-up.

**Results:**

Out of 162 recruited, 152 treatment-naïve patients contributed 144.1 person-years of observation (PYO). 86 (57%) of them were men and their median age was 32 years. 24 patients died, making the overall mortality rate 16.7 per 100 PYO. The highest death rate occurred in the first month of treatment. Compared to the first month, mortality declined by 9-fold after the 18^th ^week of follow-up. Being in WHO clinical stage IV and having TLC<= 750/mcL were independent predictors of death. Haemoglobin (HGB) <= 10 g/dl and TLC<= 1200/mcL at baseline were not associated with increased mortality. Body mass index (BMI) <= 18.5 kg/m2 at baseline was associated with death in univariate analysis. Weight loss was seen in about a third of patients who survived up to the fourth week, and it was associated with increased death. Decline in TLC, HGB and BMI was associated with death in univariate analysis only.

**Conclusion:**

The high mortality rate seen in this cohort was associated with advanced disease stage and very low TLC at presentation. Patients should be identified and treated before they progress to advanced stages. The underlying causes for early death in patients presenting at late stages should be investigated.

## Background

In resource-poor countries, access to antiretroviral therapy (ART) has improved during the last years and mortality rates among treated patients have declined substantially [1,2]. However, compared to patients in high-income countries, patients in resource-poor countries are at higher risk of death in the early months of treatment [3]. To avoid such early deaths, we need to identify possible risk factors and potential causes of death. This requires good laboratory setup, trained personnel, and uninterrupted supply of drugs and laboratory reagents. Unfortunately, despite the current efforts to expand treatment in resource-poor settings, lack of satisfactory laboratories continues to be a challenge [4]. Even if we manage to get the equipment to such settings, as we do now in many urban settings in Africa, it is often difficult to find trained personnel, the electricity supply is unreliable, and the cost of laboratory reagents is expensive [5]. To make ART available to people living in areas with only basic laboratory setup, we need to look for simplified ways of doing this.

Earlier studies have shown that the total lymphocyte count (TLC), haemoglobin (HGB), body mass index (BMI), body weight and other simple laboratory and clinical markers predict mortality in HIV infected patients and thus can be used to identify patients at risk of dying [6-11]. However, most of these studies were done in untreated patients. Few studies have evaluated the usefulness of such simple markers as predictors of clinical events in patients receiving highly active antiretroviral therapy (HAART) [6].

In Ethiopia, we treated and followed patients using the WHO clinical stage and the TLC as criteria for beginning treatment [12]. The objective of this study was to identify predictors of early death in Ethiopian patients treated with HAART in a setting with only basic laboratory setup.

## Methods

### Study setting

We did this study at Arba Minch Hospital in southern Ethiopia. Arba Minch Hospital is a district hospital located 500 km south of Addis Ababa. The hospital serves about 1.5 million people with capacity of 158 beds. Since the early 1990s, the hospital has been delivering HIV counselling and testing services. In 2002, in preparation to start ART, we trained staff, renovated rooms, designed patient record forms and installed basic laboratory equipment. In January 2003, we started adult HIV clinic. A medical doctor, assisted by two others, headed the clinic. Other members of the team included five nurse counsellors, two laboratory technicians, two community agents, and a data clerk. The health workers were permanent employees of the hospital. The community agents and the data clerk were recruited through a public notice. These were secondary school leavers with previous record of anti-HIV activities in the community. Familiarity with data management and computer literacy were additional criteria for the data clerk. All members of the team completed the necessary national courses.

We started ART in August 2003 as one of the first public hospitals to start ART in Ethiopia. Prior to that, patients were treated only for opportunistic infections. We previously reported the pattern of disease progression among untreated patients [13], and the results comparing the clinical outcome before and after treatment with HAART [14]. Here, we report the outcome of the first consecutive patients who were treated with HAART at our clinic between August 2003 and January 2005 and followed through 9 August, 2005.

### Patient flow, treatment and follow-up

A nurse referred all HIV infected adult patients (age >= 15 years) to a medical doctor following a standard counselling and testing procedure [15] and after recording socio-demographic information, body weight, height and test results in a standardized patient record form. The doctor did standard clinical examination and staged patients according to the WHO clinical staging system [16].

Following clinical staging, complete blood cell count (CBC) was measured in all patients using automated haematology analyser (Sysmex Kx-21, Sysmex Corporation, Kobe, Japan). CD4 machines were not available. In those with indication for HAART, we did liver and renal function tests using a semi-automatic photometer (Photometer 5010, Riele, version 3.0) at baseline and then regularly according to the national treatment guideline [12].

According to the guideline, only symptomatic patients (WHO stage II-IV) were eligible for treatment. In stage II, the TLC<= 1200/mcL was used as an additional criterion. Patients were treated on a first-come first-served basis. Triple combinations of stavudine (d4T), lamivudine (3TC), nevirapine (NVP), zidovudine (ZDV), and efavirenz (EFV) were available. d4T/3TC/NVP was the first-line combination of choice. EFV was reserved for patients in their intensive phase of antituberculosis therapy. Drugs were refilled every 4 weeks. At each visit, we measured body weight. CBC was repeated 4 weeks later and then every 12 weeks.

We assigned unique identification numbers to each newly started patient. The data clerk then recorded the information and notified the community agents. Every month, the community agents visited the patients in their homes and reported the status of each patient to the data clerk and to the doctor. Death was the main outcome variable, and all non-accidental deaths were considered HIV-related. Specific causes of death were not determined. Also, the community agents reported if the patients were lost, transferred or stopped treatment.

Patients were censored on the date of any one of the following events, whichever occurred first: (i) if the patient was lost to follow-up, the date of the last contact the patient had with the community agent (ii) if the patient was transferred to another health institution, the date of transfer, (iii) if the patient stopped treatment, the last date of drug re-supply plus two months, and (iv) if the patient was alive and on treatment at the end of the follow-up, August 9 2005.

### Statistical analysis

We used the Kaplan-Meier and the Cox-regression survival analysis techniques to identify predictors of death. We included the baseline WHO clinical stage, TLC, BMI, and HGB as potential predictors. TLC, BMI, and HGB were treated as dichotomous variables at clinically useful cut-off points as described elsewhere [13,14]. Also, we calculated the magnitude and direction of change in HGB, body weight, BMI, and TLC values at the first and second follow-up visits. Then we dichotomized the resulting change in the laboratory and clinical values at two cut-off points: (i) [no change or decrease] versus [increase]; (ii) [decrease] versus [no change or increase]. Thus we evaluated changes in HGB, body weight, BMI and TLC from baseline to the first and second visits as potential predictors of death.

In the multivariate Cox regression model, we included only those variables significantly associated with death in univariate analysis. We included interaction terms in multivariate models to check for multicolinearity. Results were presented as hazard rations (HR) with 95% confidence intervals (95% CI). We used the Kaplan-Meier curves to highlight important features and the Log-rank test was used to test for statistical significance.

We calculated mortality rates for each phase of the follow-up: (i) from baseline to the first follow-up visit, (ii) from baseline to the 2^nd ^follow-up visit, (iii) from the first follow-up to the second follow-up visit, (iv) from the first follow-up visit to the end of the study, (v) from the second follow-up visit to the end of the study, and (vi) from baseline to the end of the study. Mortality rates were described as number of deaths per 100 person-years of observation (PYO). Then we calculated mortality rate ratios for each phase of the follow-up. We used SPSS version 14.0 (SPSS Inc., Chicago, IL, USA) for data analysis. All statistical tests were two-sided and P <0.05 was considered statistically significant.

### Ethical considerations

The National Ethics Review Committee in Ethiopia and the Regional Committee for Medical Research Ethics in Western Norway approved the study protocol. All patients gave informed consent before HIV testing and for taking part in the study.

## Results

### Baseline results

**Figure 1:**
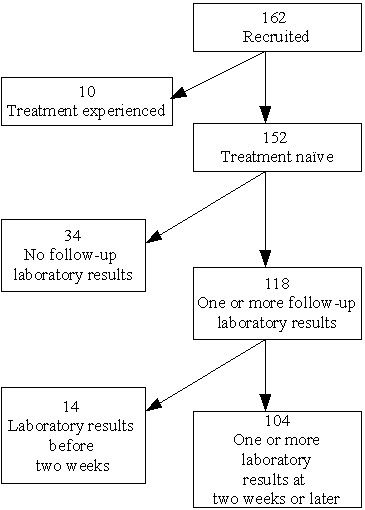
**Cohort profile**. 34 patients had no follow-up laboratory records at 4 weeks. In 12 of the 34 patients, end point events including eight deaths occurred before the planned first visit.

We enrolled and treated 162 patients between August 2003 and January 2005. Ten treatment-experienced patients were excluded from analysis. Out of the 152 treatment-naïve patients, 86(57%) were men and 66 (43%) were women. Their median age was 32 years [interquartile range (IQR), 28.5–40]. 34 patients with no follow-up laboratory records were included in the baseline analysis but excluded from the follow-up analysis which included change in specific laboratory values as predictors. In 12 of the 34 patients, end point events including eight deaths occurred before the planned first visit. In the remaining 22 patients, the events occurred after the planned first visit (5 died within 5–12 weeks of starting treatment, 1 stopped, 1 died after 12 weeks, 15 were still on follow-up). 68% (104/152) of the treatment-naïve patients were in WHO stage III, 23% (35/152) in stage IV, and 9% (13/152) in stage II. 72 patients (47%) started d4T/3TC/NVP, 45(30%) d4T/3TC/EFV, 23 (15%) ZDV/3TC/NVP and 12 (8%) patients received ZDV/3TC/EFV. The general profile of the cohort is shown in Figure 1.

The median (range) HGB was 9.1 g/dl (4.9–12.7); 8.5 g/dl (4.4–13.9) for women and 9.3 g/dl (3.7–15.3) for men (P = 0.078). 67% (102 of 152 patients) had HGB<= 10 g/dl at presentation. The median (range) TLC count was 1000 cells/mcL (200–4800) and similar among men and women (P = 0.966). The median (range) weight was 48 kg (32–83)] for women and 53 kg (35–74)] for men. Their median (range) BMI was 18.3 (13.0–27.8); 18.6 (13.5–27.8) in women and 18.2 (13.0–24.6) in men.

### Follow-up results

#### Change in selected clinical and laboratory values

The median time to the first follow-up visit was 4 weeks (IQR, 4–5), and the second visit was at a median time of 18 weeks (IQR, 16–22). The median (95% range) weight gain was 1 kg (-7.4, 7.7) and 3 kg (-8, 14.7) at the first and second follow-up visits, respectively. The HGB declined by a median of 0.20 g/dl (95% range;-5.97, 4.50) at week 4, while it increased by a median of 0.60 g/dl (95% range; -6.94, 3.90) from baseline to week 18. The TLC increased by a median (95% range) of 100 cells/mcL (-1125, 2180) and 300 cells/mcL (-2025, 1700) at weeks 4 and 18, respectively. When described as dichotomous changes, 34% (31/91) of the patients lost weight from baseline to the first visit. Similarly, decline in TLC was recorded in 35% (35/101) of the patients at week 4.

#### Mortality and its predictors

As of August 9 2005, 152 treatment naïve patients contributed 144.1 PYO. 24 patients died during a median follow-up of 55 weeks (IQR; 29–71 weeks), making the overall mortality rate 16.7 per 100 PYO (24 deaths/144.1 PYO). 33% (8/24) and 75% (18/24) of the deaths occurred within 4 and 18 weeks, respectively after starting treatment. Thus the mortality rates for the first 4 and 18 weeks were 57.5 (8/13.9 PYO) and 37.3 (18/48.3) per 100 PYO, respectively. After the 4^th ^week, mortality declined to 12.3 per 100 PYO (16 deaths/130.2 PYO). The mortality further declined to 6.3 per 100 PYO (6 deaths/95.8 PYO) after the 18^th ^week of follow-up. Table 1 describes mortality rates for different phases of the follow-up.

**Table 1:** Mortality rates and rate ratios by treatment phase, Arba Minch Hospital, 2006

Treatment phase	Deaths	PYO	Rate	Rate ratio (95%CI)
0–4 weeks	8	13.9	57.5	1
0–18 weeks	18	48.3	37.3	0.7 (0.3–1.7)
4–18 weeks	10	34.4	29.1	0.5 (0.2–1.2)
4 weeks-end of study	16	130.2	12.3	0.2 (0.1–0.5)
18 weeks-end of study	6	95.8	6.3	0.1 (0.03–0.3)
Overall	24	144.1	16.7	0.3 (0.2–0.4)

PYO = Person-Years of Observation 95%CI = 95% Confidence Interval

In univariate analysis, TLC <= 1200/mcL and HGB <= 10 g/dl at baseline were not associated with mortality (P > .1 for both). The BMI<= 18.5 kg/m2 was significantly associated with overall mortality only in univariate analysis [HR (95%CI) = 2.9 (1.04–8.01), P = 0.042]. TLC<= 750/mcL at baseline was the strongest predictor of overall mortality [HR (95%CI) = 3.4 (1.5–7.6), while baseline WHO stage IV was the strongest predictor of death in the first 4 weeks [HR (95%CI) = 9.2 (1.8–45.8), P = 0.007]. Tables 2 and 3 summarize the Cox regression analyses.

**Table 2:** Predictors of overall survival according to Cox regression analyses, Arba Minch Hospital, 2006

Factor	Crude HR (95%CI)	P-value	Adjusted HR (95%CI)	P-value
TLC <= 750 vs. >750/mcL	3.6 (1.5–8.1)	0.002	3.6 (1.4–9.6)	0.009
WHO stage IV vs. II-III	3.7 (1.6–8.5)	0.002	2.7 (1.01–7.3)	0.047
BMI <= 18.5 vs. >18.5 kg/m2	2.9 (1.04–8.0)	0.042	2.7 (0.9–7.4)	0.062
HGB <= 10 g/dl vs. >10 g/dl	2.5 (0.8–7.4)	0.094	--	--
TLC <= 1200 vs. >1200/mcL	2.3 (0.8–6.8)	0.130	--	--

HGB = Haemoglobin TLC = total lymphocyte count BMI = Body Mass Index WHO = World Health Organization HR = Hazard Ratio 95%CI = 95% Confidence Interval

**Table 3:** Hazard ratios (HR) of death in the first four weeks of treatment, Arba Minch Hospital, 2006

Factor	Crude HR(95%CI)	P-value	Adjusted HR (95%CI)	P-value
WHO stage IV vs. II-III	10.5 (2.1–52.4)	0.004	9.2 (1.8–45.8)	0.007
TLC<= 750 vs. >750/mcL	5.2 (1.2–21.9)	0.024	4.4 (1.05–18.5)	0.043
HGB<= 10 vs. >10 g/dl	3.5 (0.4–28.3)	0.244	--	--
TLC<= 1200 vs. >1200/mcL	3.4 (0.4–25.7)	0.282	--	--
BMI<= 18.5 vs. >18.5 kg/m2	1.5 (0.3–9.3)	0.629	--	--

HGB=Haemoglobin TLC=total lymphocyte count BMI=Body Mass Index WHO=World Health Organization HR=Hazard Ratio 95%CI = 95% Confidence Interval

**Figure 2:**
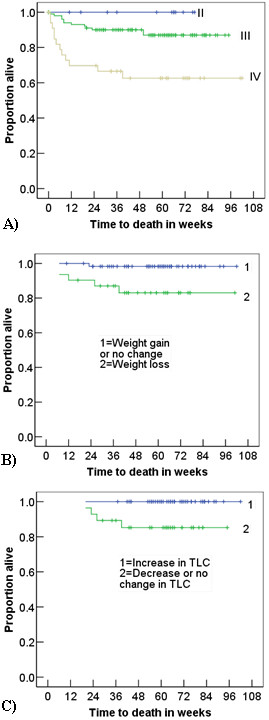
**Mortality according to disease stage, weight loss and decline in total lymphocyte count. A – Mortality according to WHO clinical staging**. Note that mortality was highest in patients with stage IV disease particularly in the first 12 weeks of treatment. **B- Kaplan-Meier survival curve showing higher mortality among patients with weight loss**. Since weight was measured at about 4 weeks of treatment, it does not show earlier deaths. Rather it shows deaths that occurred after 12 weeks of treatment. Note that the time-axis represents the time from baseline to death. **C- Change in total lymphocyte count and mortality**. This figure shows the higher mortality among patients with no change or decrease in total lymphocyte count from baseline to week 18.

Out of the 104 patients with at least one follow-up CBC, 9 patients died over a median period of 61 weeks from baseline (IQR; 42–74 weeks). Weight loss at week 4 was associated with shorter survival compared with no change or weight gain (Log-rank; P = 0.009). Negative or no change in the TLC at week 4 was not associated with mortality (Log rank; P = 0.532), but negative or no change from baseline to week 18 was significantly associated with death (Log-rank; P = 0.008). Figure 2 shows the survival estimates. Decline in BMI from baseline to the first follow-up visit was also associated with poor prognosis (Log-rank; P = 0.012). Patients with decline in HGB value at the first follow-up visit had worse prognosis than those who had no or positive change in HGB level (Log-rank; P = 0.040). In Cox regression analysis none of these variables were associated with mortality (data not shown).

## Discussion

In this cohort, mortality was highest during the first month of treatment, about 9-fold higher compared to mortality after the 18^th ^week of follow-up. The WHO clinical stage was the strongest predictor of death in the first month. The TLC<= 750/mcL, but not the TLC<= 1200/mcL, was strongly associated with overall mortality in this cohort. A third of the patients who survived up to the fourth week lost body weight, and weight loss was associated with increased death after the first month. Also, decline in the TLC from baseline to the 18^th ^week predicted death.

The findings have practical implications for managing HIV infected patients in resource-limited settings. The high early mortality in patients with advanced disease implies the need for starting treatment earlier. This requires early diagnosis of HIV infection through improved counselling and testing practices. For patients who present at advanced stage, more frequent contact with the patient may help prevent potentially treatable conditions such as mycobacterial infections [17]. The other striking finding in this study is the very low TLC which predicted mortality. Although the recommended TLC for initiating treatment is below 1200/mcL, this failed to identify patients at immediate risk of dying. This highlights the need for paying closer attention to patients with very low levels of TLC. Weight loss is still a common problem in patients treated with HAART, and its presence should alert further search for underlying causes.

The pattern of mortality observed in our cohort is consistent with findings from other resource-poor settings. In Senegalese patients, mortality in the first year of follow-up was 12.5 per 100 PYO, and decreased to 0.9 per 100 PYO at five years [17]. The overall mortality rate of 16.7 per 100 PYO in our cohort is comparable to the Senegalese cohort in the first year. In South Africa, Lawn et al reported a regular decline in mortality from 35.7 during pre-treatment follow-up to 17.5 per 100 PYO during the first month of treatment [18]. After 6–9 months, the mortality in the South African cohort was lower by about 13-fold. In our cohort, although the first-month mortality was much higher at 57.5 per 100 PYO, the 9-fold decline observed after 18 weeks of follow-up was comparable. Inclusion of only symptomatic patients (stage II-IV) in our cohort might have contributed to the observed difference in mortality. A pooled analysis of data from low-income and high-income countries showed that mortality was higher in low-income countries by 4.3-fold during the first month, but the reported mortality rate was low at 5.8% in the first year [3]. As stated by the authors, exclusion of substantial proportion of patients from the analysis might have resulted in a biased estimate in their report.

A few studies have evaluated the prognostic value of the TLC in treated patients [6,17]. In the Senegalese patients, TLC<= 1200/mcL was associated with increased risk of death. However, when adjusted for anaemia it did not predict mortality [17]. In our cohort, TLC<= 750/mcL, but not TLC<= 1200/mcL, was an independent predictor of death. Since we treated only symptomatic patients whose median TLC was 1000/mcL, it is likely the small number of patients with TLC >1200/mcL affected our results.

HGB and BMI are described as important prognostic markers either independently or in combination with the TLC both in untreated and in treated patients [9,17,19-22]. In our cohort, BMI<= 18.5 kg/m^2 ^was significantly associated with overall mortality in univariate analysis, but not when adjusted for TLC<= 750/mcL and WHO clinical stage. HGB<= 10 g/dl was also associated with increased overall mortality in univariate analysis only. This could be because of the few patients with HGB and BMI values above the stated thresholds.

As shown by others, we found that the WHO clinical stage was an independent marker of mortality in patients treated with HAART [17,18]. Compared to the TLC, the WHO stage was a stronger predictor of death in the first month of treatment. While the ultimate goal should be to treat patients before they progress to such advanced stages, doctors in new settings will continue to treat such patients because of poor testing practices in Africa [23]. Therefore, careful documentation of the patient's disease stage will be helpful in identifying patients who need more intense follow-up. Improving the counselling and testing practices should be viewed as a more sustainable strategy.

Despite being an easily recognizable and common condition, weight loss has not been studied among resource-poor adult patients treated with HAART. The prognostic value of weight loss has been well documented in developed countries [24-26]. In one study, about a third of the patients treated with HAART had weight loss, as is in our patients, and it remained the most important prognostic marker [24]. Poor nutritional intake, metabolic disturbances because of drugs, and infectious conditions such as tuberculosis could be possible contributing reasons [27].

The main limitation of this study is the small number of patients and events. Despite the clear trend towards increased mortality in patients with BMI<= 18.5 kg/m^2 ^and HGB<= 10 g/dl, we were not able to determine whether these were independent factors. The same is true for the TLC <= 1200/mcL. Therefore, further follow-up of the cohort with inclusion of more patients should give answers to these questions.

## Conclusion

We found a very high mortality rate in this cohort especially during the first month of treatment. The prognosis was worse in patients with advanced disease and with TLC<= 750/mcL. This highlights the need for identifying and treating patients early through improved counselling and testing strategies. Moreover, the underlying mechanisms for the weight loss observed in a third of our patients should be investigated and, if found, interventions should be planned for.

## Competing interests

The author(s) declare that they have no competing interests.

## Authors' contributions

DJ and BL designed the study. DJ, AE and YH recruited and followed the patients. DJ and BL analysed the data. All the authors contributed to the drafting and approval of the manuscript.

## Pre-publication history

The pre-publication history for this paper can be accessed here:


